# Genetic deletion of calcium/calmodulin-dependent protein kinase type II delta does not mitigate adverse myocardial remodeling in volume-overloaded hearts

**DOI:** 10.1038/s41598-019-46332-3

**Published:** 2019-07-08

**Authors:** Belal A. Mohamed, Manar Elkenani, Joanna Jakubiczka-Smorag, Eric Buchholz, Sabrina Koszewa, Dawid Lbik, Moritz Schnelle, Gerd Hasenfuss, Karl Toischer

**Affiliations:** 1 Department of Cardiology and Pneumology, Georg-August-University, Göttingen, Germany; 2 DZHK (German Centre for Cardiovascular Research), partner site Göttingen, Göttingen, Germany; 3 Department of Medical Biochemistry and Molecular Biology, Faculty of Medicine, Mansoura University, Mansoura, Egypt; 4 King’s College London British Heart Foundation Centre of Excellence, Cardiovascular Division, London, United Kingdom; 5 Institute for Clinical Chemistry, University Medical Center Göttingen, Göttingen, Germany

**Keywords:** Cardiology, Heart failure

## Abstract

Calcium/calmodulin-dependent protein kinase type II delta (CaMKIIδ), the predominant CaMKII isoform expressed in the heart, has been implicated in the progression of myocardial infarction- and pressure overload-induced pathological remodeling. However, the role of CaMKIIδ in volume overload (VO) has not been explored. We have previously reported an activation of CaMKII during transition to HF in long-term VO. Here, we address whether CaMKIIδ is critically involved in the mortality, myocardial remodeling, and heart failure (HF) progression in response to VO. CaMKIIδ knockout (δ-KO) and wild-type (WT) littermates were exposed to aortocaval shunt-induced VO, and the progression of adverse myocardial remodeling was assessed by serial echocardiography, histological and molecular analyses. The mortality rates during 10 weeks of VO were similar in δ-KO and WT mice. Both genotypes displayed comparable eccentric myocardial hypertrophy, altered left ventricle geometry, perturbed systolic and diastolic functions after shunt. Additionally, cardiomyocytes hypertrophy, augmented myocyte apoptosis, and up-regulation of hypertrophic genes were also not significantly different in δ-KO versus WT hearts after shunt. Therefore, CaMKIIδ signaling seems to be dispensable for the progression of VO-induced maladaptive cardiac remodeling. Accordingly, we hypothesize that CaMKIIδ-inhibition as a therapeutic approach might not be helpful in the context of VO-triggered HF.

## Introduction

Adverse myocardial remodeling that precedes cardiac muscle dysfunction is characterized by a myriad of molecular, structural, and functional changes in response to hemodynamic overload and/or myocardial injury^1^. Hemodynamic overload can be classified into pressure overload (PO) and volume overload (VO), triggering concentric and eccentric cardiac hypertrophy, respectively. Mitral and aortic valve regurgitation (MR and AR, respectively) is the primary causes of VO that, in contrast to PO, exhibits an early adaptive phenotype with late onset transition to HF^2,3^. Accordingly, it is not surprising that some drugs that efficiently attenuate PO-induced cardiac remodeling do not exhibit similar beneficial effects in VO^4^. Hence, understanding the pathophysiology of VO is a mandatory step for developing therapeutics to treat VO-induced congestive heart failure (HF), a clinical condition resistant to standard therapeutic strategies^5^.

A will reproducible model to study the pathophysiology of VO is the aorto-caval shunt, in which 3 stages of myocardial remodeling have been identified^6^: an acute phase coming from abrupt increased preload that results in increased diastolic wall stress leading to early left ventricle (LV) dilatation; a compensatory phase, characterized by sustained preload elevation that results in eccentric hypertrophy, progressive increase in LV internal diameter and preservation of wall thickness that still efficiently compensates for elevated diastolic wall stress and therefore associated with preserved systolic function; and late decompensatory phase, manifested by further chamber dilatation, continued wall thinning, systolic and diastolic dysfunction, and eventual pump failure.

A hallmark of HF development is decreased cardiac contractility that is mainly due to impaired intracellular Ca^2+^ handling^7^. Calcium/calmodulin-dependent protein kinase type II (CaMKII), a critical regulator of cardiac excitation-contraction coupling^8^, is a multifunctional serine/threonine protein kinase that is activated by Ca^2+^/calmodulin, autophosphorylation and post-translational modifications^9,10^. Four CaMKII isoforms have been described; α and β are neuron-specific, while γ and δ are ubiquitously expressed. In the heart, CaMKIIδ is the most abundant isoform, although CaMKIIγ is also expressed to a lesser extent^11^. Deletion of CaMKIIδ could avert adverse remolding in response to PO and other cardiac injuries^12–17^. However, little is known about the role of CaMKIIδ in VO. Recently, we reported an increased CaMKII activity during HF transition in wild-type (WT) mice subjected to long-term VO^2^.

To determine whether CaMKIIδ is crucial for VO-induced adverse remodeling, CaMKIIδ-knockout (δ-KO)^12^ and WT littermates were exposed to long-term VO by surgical aortocaval shunt (shunt) and cardiac structure and function were assessed by serial echocardiography and molecular analyses. Here we showed that CaMKIIδ deletion is not sufficient to avert or attenuate the adverse myocardial remodeling and HF progression in mice undergoing chronic VO.

## Results

### Comparable mortality rates in δ-KO and WT mice after shunt

**Figure 1:**
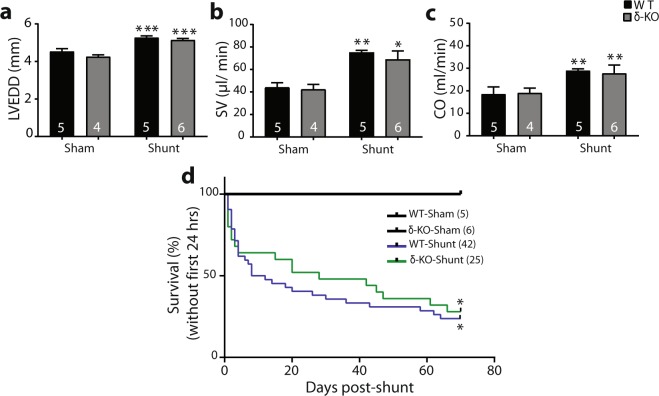
CaMKIIδ deletion does not improve mortality rates post-shunt. (**a–c**) Comparable left ventricle dilatation, stroke volume and cardiac output at 1-week post-shunt in both genotypes. (**d**) Kaplan-Meier survival curves depicting the effect of CaMKIIδ deletion on 10-week overall survival in VO. ^*^p < 0.05, ^**^p < 0.01, ^***^p < 0.001 *vs*. corresponding sham, 1-way ANOVA with Bonferroni post-test (a-c) and log-rank test (d). Numbers within columns indicate mice.

Because CaMKII activity is increased after long-term shunt^2^, we hypothesized that CaMKII is critical for transition to HF in responses to VO. To test this concept, we used δ-KO mice^12^, lacking the dominant cardiac δ isoform. Consistent with previous studies^12,14^, the δ-KO mice were viable, fertile, developed normally and showed no significant difference in cardiac function and structure when compared with WT littermates (Table S1). Homozygous δ-KO mice and WT littermates underwent experimental VO induced by surgical shunt. The LV end-diastolic diameter (LVEDD), stroke volume (SV), and cardiac output (CO) were significantly, but similarly, increased at 1-week post-shunt in both genotypes, indicating comparable VO (Fig. 1a–c). The mortality rates up to 10 weeks after shunt did not differ between WT and δ-KO mice (WT-shunt vs. δ-KO-shunt, ≈76% (32 out of 42) *vs*. ≈72% (18 out of 25) (Fig. 1d).

### Progressive contractile dysfunction and dilation in δ-KO and WT after shunt

**Figure 2:**
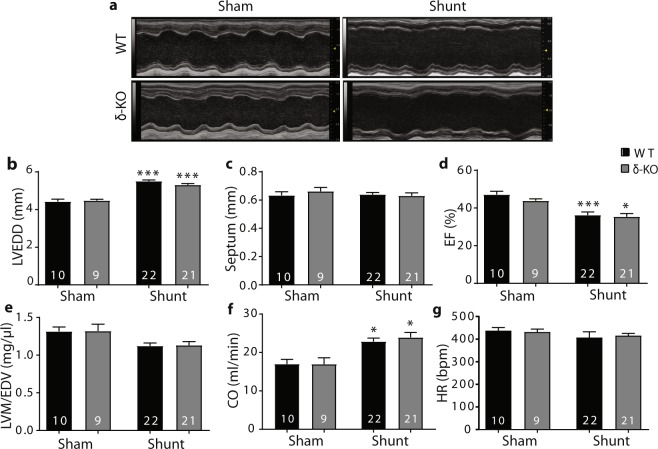
Altered myocardial geometry and systolic impairment are not different in δ-KO and WT hearts at 12 weeks of VO. (**a**) Representative M-mode echocardiogram. (**b–g)** Parameters of cardiac structure and function. Data are presented as mean ± SEM. ^*^p < 0.05, ^***^p < 0.001 *vs*. corresponding sham, 1-way ANOVA with Bonferroni post-test. Numbers within columns indicate mice. bpm, beats/minute; CO, cardiac output; EF, ejection fraction; HR, heart rate; LVEDD, left ventricular end-diastolic diameter; LVM/EDV, left ventricular mass-to-end-diastolic volume.

To evaluate the effects of CaMKIIδ deletion on the development of cardiac remodeling and failure after chronic VO, LV geometry and function were evaluated using serial echocardiography. Compared to sham mice, both δ-KO and WT shunt mice exhibited early pronounced myocardial dilatation and late contractile failure, but unchanged septum wall thickness, indicating an eccentric hypertrophy (Fig. 2a–d; Table 1). However, there were no significant differences between shunt-WT and -δ-KO mice. According to Laplace’s law, LV mass-to-end-diastolic volume (EDV) ratio is inversely proportional to cardiac wall stress. LV mass-to-EDV ratio, calculated via echocardiography, was similarly decreased at 12 weeks post-shunt (≈17% decrease in shunt vs. sham, Fig. 2e), indicating similar increases in cardiac wall stress in both genotypes. The heart rates were not different amongst all four groups (Fig. 2g).

**Table 1:** Echocardiographic parameters in δ-KO and WT mice at 4 and 16 weeks after surgeries.

	Sham		4 weeks post-shunt		16 weeks post-shunt	
	WT (*n* = 9)	δ-KO (*n* = 10)	WT (*n* = 24)	δ-KO (*n* = 18)	WT (*n* = 22)	δ-KO (*n* = 16)
HR (bpm)	409.9 ± 12.9	432.1 ± 16.5	418.7 ± 18.9	421.5 ± 18.8	404.0 ± 5.3	427.6 ± 12.7
LVESD (mm)	3.72 ± 0.08	3.63 ± 0.15	4.34 ± 0.07^**^	4.27 ± 0.06^***^	4.36 ± 0.06^***^	4.52 ± 0.15^**^
LVEDD (mm)	4.66 ± 0.10	4.55 ± 0.12	5.65 ± 0.07^***^	5.43 ± 0.08^***^	5.36 ± 0.06^***^	5.49 ± 0.13^**^
Septum (mm)	0.64 ± 0.01	0.65 ± 0.02	0.68 ± 0.02	0.68 ± 0.02	0.64 ± 0.01	0.66 ± 0.02
LVM/EDV	1.27 ± 0.06	1.29 ± 0.05	1.16 ± 0.03	1.17 ± 0.03	1.10 ± 0.02^*^	1.07 ± 0.04^*^
FS (%)	20.53 ± 1.33	20.89 ± 2.39	23.15 ± 1.00	21.23 ± 1.23	16.65 ± 0.59^*^	15.44 ± 1.33^*^
EF (%)	41.79 ± 2.27	42.33 ± 4.10	45.39 ± 1.61	42.39 ± 2.03	35.67 ± 1.35^*^	32.03 ± 2.57^*^
CO (ml/min)	16.48 ± 0.72	17.69 ± 1.40	24.43 ± 0.96^*^	23.90 ± 2.01^*^	23.51 ± 0.58^*^	24.77 ± 1.67^*^

Data are expressed as mean ± SEM. ^*^p < 0.05, ^**^p < 0.01, ^***^p < 0.001 *vs*. corresponding sham, 1-way ANOVA with Bonferroni post-test. bpm beats per minute; CO, cardiac output; EF, ejection fraction; FS, fractional shortening; HR, heart rate; LVEDD, left ventricular end-diastolic diameter; LVESD, left ventricular end-systolic diameter; LVM/EDV, left ventricle mass-to-end-diastolic volume; WT, wild-type.

### Strain rate imaging revealed functional deterioration after shunt in both genotypes

**Figure 3:**
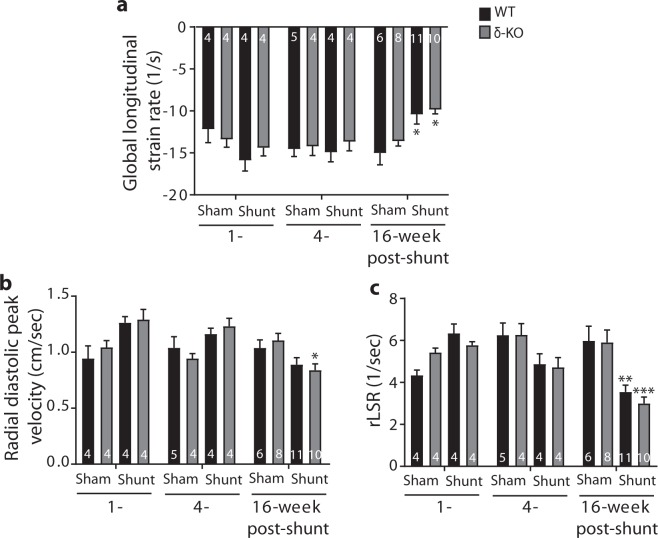
Speckle tracking echocardiography reveals systolic and diastolic dysfunction in δ-KO and WT littermates after shunt. (**a)** Mean global longitudinal strain rate. (**b)** Radial diastolic peak velocity. (**c)** Average reverse longitudinal strain rate (rLSR). Data are expressed as mean ± SEM. ^*^p < 0.05, ^**^p < 0.01, ^***^p < 0.001 *vs*. corresponding sham, 1-way ANOVA with Bonferroni post-test. Numbers within columns indicate mice.

We used high-frequency speckle tracking echocardiography to quantify myocardial strain. Consistent with progressive deterioration of EF, the global longitudinal strain rate was significantly reduced in both genotypes at 16 weeks after shunt, indicating a progressive systolic dysfunction (Fig. 3a). We also used speckle tracking to address whether CaMKIIδ loss would affect diastolic function post-shunt. The peak reverse longitudinal strain rate and radial diastolic peak velocity were markedly decreased in both genotypes at 16 weeks post-shunt, suggesting a comparable diastolic dysfunction (Fig. 3b,c).

### CaMKII deletion did not improve the histological and molecular signatures of 12 weeks VO-induced myocardial remodeling

Further analyses were performed at 12 weeks post-shunt, a time point corresponding to early deterioration of systolic function and hence early transition to HF. There was no difference in body weight among the 4 groups (Table 2). Being a biventricular VO model, both shunt groups displayed left and right ventricular hypertrophy compared to sham mice. However, no marked differences were found between the shunt-operated WT and δ-KO mice (Table 2). Moreover, lung weight-to-tibia length ratio was similarly increased in both genotypes after shunt, indicating a comparable pulmonary edema in the context of congestive HF (Table 2).

**Table 2:** Morphometric parameters in δ-KO and WT mice at 12 weeks after surgeries.

	WT-sham (*n* = 6)	δ-KO-sham (*n* = 5)	WT-shunt (*n* = 11)	δ-KO-shunt (*n* = 9)
BW (g)	28.80 ± 1.07	28.44 ± 0.72	29.22 ± 1.12	29.37 ± 1.02
HW/BW (mg/g)	4.74 ± 0.11	5.20 ± 0.20	7.15 ± 0.30^***^	7.35 ± 0.29^***^
HW/TL (mg/ml)	7.79 ± 0.32	8.23 ± 0.51	11.51 ± 0.59^**^	12.20 ± 0.62^**^
LVW/BW (mg/g)	3.35 ± 0.06	3.68 ± 0.16	5.07 ± 0.25^***^	5.19 ± 0.20^***^
LVW/TL (mg/mm)	5.55 ± 0.24	5.83 ± 0.42	8.19 ± 0.55^**^	8.63 ± 0.45^**^
RVW/BW (mg/g)	0.89 ± 0.05	0.93 ± 0.04	1.32 ± 0.09^**^	1.41 ± 0.07^*^
RVW/TL (mg/mm)	1.45 ± 0.14	1.50 ± 0.08	2.10 ± 0.11^**^	2.34 ± 0.14^**^
LungW/BW (mg/g)	5.04 ± 0.14	5.18 ± 0.11	5.87 ± 0.25^*^	6.21 ± 0.20^**^
LungW/TL (mg/mm)	7.82 ± 0.26	8.39 ± 0.09	9.55 ± 0.35^**^	10.41 ± 0.44^**^
KidW/BW (mg/g)	6.51 ± 0.19	7.08 ± 0.49	6.35 ± 0.29	6.33 ± 0.41
KidW/TL (mg/mm)	10.80 ± 0.68	11.21 ± 0.93	10.27 ± 0.63	10.44 ± 0.54

Data are expressed as mean ± SEM. ^*^p < 0.05, ^**^p < 0.01, ^***^p < 0.001 *vs*. corresponding sham, 1-way ANOVA with Bonferroni post-test. BW, body weight; HW/BW, heart weight-to-body weight; HW/TL, heart weight-to-tibia length; KidW/BW, kidney weight-to-body weight; KidW/TL, kidney weight-to-tibia length; LungW/BW, lung weight-to-body weight; LungW/TL, lung weight-to-tibia length; LVW/BW, left ventricular weight-to-body weight; LVW/TL, left ventricular weight-to-tibia length; RVW/BW, right ventricular weight-to-body weight; RVW/TL, right ventricular weight-to-tibia length; WT, wild-type.

**Figure 4:**
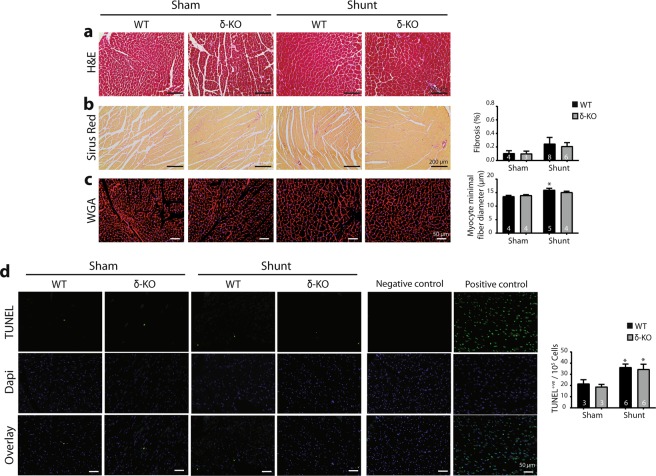
Cardiomyocytes hypertrophy and induced apoptosis occurs in δ-KO and WT hearts at 12 weeks of VO. (**a)** Representative cardiac transverse sections stained with hematoxylin and eosin (H&E). (**b)** Picrosirius red stained sections are illustrated (left panels) and quantification of fibrosis (right panel). (**c)** Stained cardiac sections with wheat germ agglutinin (WGA, left panels) and quantification of myocytes minimal fiber diameter (right panel). (**d)** Representative photomicrographs showing TUNEL staining (left panels) and quantification of TUNEL-positive cells (right panel). Positive (DNase I treatment) and negative (without TUNEL enzyme) controls were also shown. Data are presented as mean ± SEM. ^*^p < 0.05 *vs*. corresponding sham, 1-way ANOVA with Bonferroni post-test. Numbers within columns indicate mice.

Overall morphology showed no apparent differences between shunt-operated WT and δ-KO hearts as indicated by H&E and picrosirius red staining (Fig. 4a,b). Wheat germ agglutinin-stained sections showed cardiomyocyte hypertrophy after shunt, and the cardiomyocytes minimal fiber diameter increased to a greater extent in WT (≈17%) than in δ-KO (≈9%) hearts, but the difference between shunt groups did not reach statistical significance (*p* = 0.57, Fig. 4c). We also determined the extent of apoptotic cell death after shunt by TUNEL assay. Hearts from shunt-operated mice showed a marked increase of TUNEL-positive cells as compared to sham controls, but no marked difference between WT-shunt and δ-KO-shunt could be identified (Fig. 4d).

**Figure 5:**
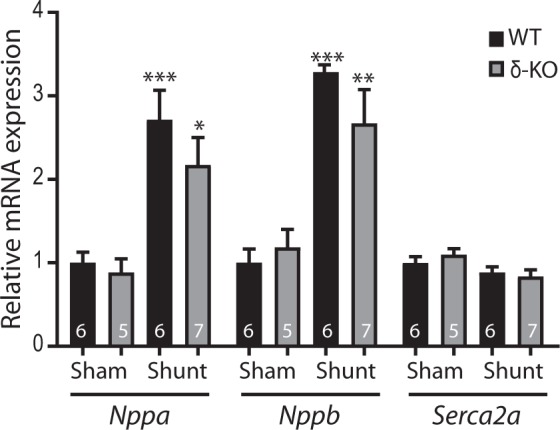
CaMKIIδ deletion does not alter the expression of cardiac stress markers at 12 weeks post-shunt. Quantitative real-time PCR analyses of fetal cardiac genes expression. Data are mean ± SEM. ^*^p < 0.05, ^**^p < 0.01, ^***^p < 0.001 *vs*. corresponding sham, 1-way ANOVA with Bonferroni post-test. Numbers within columns indicate mice. *Nppa*, natriuretic peptide type A; *Nppb*, natriuretic peptide type B; *Serca2a*, sarcoplasmic reticulum Ca^2+^-ATPase-2a.

At the molecular level, shunt-subjected δ-KO and WT mice displayed comparable reactivation of the fetal cardiac gene program, including natriuretic peptide type A (*Nppa*) and natriuretic peptide type B (*Nppb*), and a trend towards down-regulation of sarcoplasmic reticulum Ca^2+^-ATPase-2a (*Serca-2a*) (Fig. 5). Thus, CaMKIIδ deletion does not appear to significantly avert maladaptive cardiac remodeling during chronic VO.

### Equal activation of CaMKII in δ-KO and WT hearts after shunt

**Figure 6:**
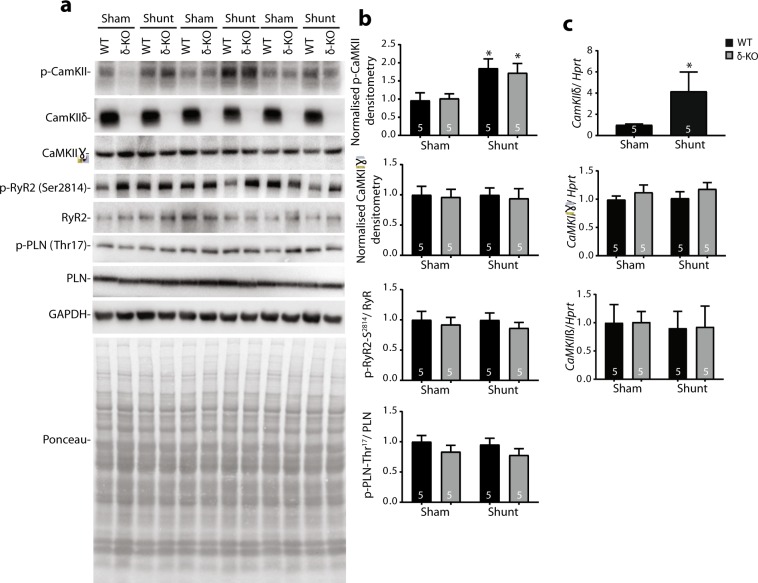
Analysis of CaMKII targeted proteins in δ-KO and WT hearts at 12 weeks of VO. (**a)** Representative Immunoblots and Ponceau-stained blots from left ventricles of δ-KO and WT hearts (Uncropped blots are shown in Fig. S1). (**b)** Bands intensity was normalized to Ponceau staining. Analysis per heart was performed in duplicates. (**c)** mRNA expression of *CamkII* isoforms. Data are mean ± SEM. ^*^p < 0.05 *vs*. corresponding sham, 1-way ANOVA with Bonferroni post-test. Numbers within columns indicate mice.

To address whether CaMKII activation would be abated in δ-KO hearts, we used the commercially available anti-phospho-CaMKII antibody, which unfortunately does not distinguish the different CaMKII isoforms. Consistent with our previous results^2^, CaMKII activity, inferred from phospho-CaMKII levels, increased in WT hearts at 12 weeks post-shunt. Surprisingly, the CaMKII phosphorylation were also enhanced in δ-KO after shunt to nearly the same level seen in WT hearts, although complete loss of CaMKIIδ in cardiac extracts of δ-KO mice was confirmed (Fig. 6a,b). We also measured the phosphorylation of the CaMKII target sites, phospholamban (PLB)-Thr17 and ryanodine receptor (RyR2)-Ser2814, by Western blot analyses. Both phosphorylation sites were slightly but not significantly reduced in δ-KO *vs*. WT hearts (Fig. 6a,b). Immunoblotting analysis revealed no difference in CaMKIIγ protein levels between δ-KO and WT hearts, in both sham and shunt mice (Fig. 6a,b).

Consistent with protein data, *CamkIIδ* mRNA was markedly induced in WT hearts after shunt (Fig. 6c). To address whether the other CaMKII isoforms could compensate for CaMKIIδ deletion, we quantified their expression levels. Whereas *CamkIIα* was not detectable, *CamkIIβ* and *CamkIIɣ* were not differentially expressed in both genotypes in sham and shunt (Fig. 6c).

## Discussion

The present study investigated the relevant role of the predominant cardiac CaMKII isoform, CaMKIIδ, in the setting of VO and showed that: [1] VO-triggered adverse myocardial remodeling was not attenuated upon CaMKIIδ deletion. Consistently, mortality rates after shunt were similar in δ-KO and WT mice. [2] Despite efficient CaMKIIδ deletion, comparable CaMKII activity was detected in δ-KO- and WT-sham that significantly but similarly increased after shunt.

Previously, we reported that the Ca^2+^ cycling in VO model is not disturbed and the myocardial remodeling, in comparison to PO, is more favorable at least in its acute phase^3,18^. While stimulation of Ca^2+^ cycling is induced in PO to increase the myofilament activation, VO is compensated by the Frank-Starling mechanism, which is Ca^2+^ cycling-independent. The changes in PO are partially and very early mediated by CaMKIIδ activation, leading to disturbed intracellular Ca^2+^ homeostasis. In contrast, VO shows no sign of early CaMKIIδ activation^3^. At later stages, disturbed Ca^2+^ cycling, as evidenced by CaMKIIδ activation and protein kinase B/AKT inactivation, are visible in the VO model and probably contribute to the HF phenotype^2^, suggesting that maintenance of AKT and/or antagonizing CaMKII signalings might be promising therapeutics to avert maladaptive remodeling in response to VO. Consistently, VO-triggered pathological remodeling is aggravated in *Akt*-KO mice^2^, and hence small molecules to retain AKT signaling might be a novel therapy for the treatment of congestive HF^19^.

However, little is known about the role of CaMKII in VO. In cardiomyocytes, CaMKII phosphorylates and regulates several key proteins involved in intracellular Ca^2+^ signaling, including PLB, RyR2 and the L-type Ca^2+^ channel^20,21^. CaMKII expression and activity increase in patients and animal models of HF^3,22,23^. CaMKIIδ inactivates histone deacetylase 4, leading to transcriptional activation of myocyte enhancer factor 2 and up-regulation of hypertrophic marker genes^24^. Consistently CaMKIIδ transgenic mice display pathological cardiac hypertrophy^25,26^, whereas genetic CaMKIIδ deletion mitigates adverse LV remodeling triggered by PO, Gαq expression, β-adrenergic stimulation, and ischemia/reperfusion^12–17^.

VO has several simultaneous events including increased adrenergic drive, cardiomyocyte oxidative stress, and an intense inflammatory response^27–29^. CamKIIδ is considered a nexus between detrimental neurohumoral activities and remodeling, and was reported to mediate inflammation and oxidative stress^30–32^. Therefore, we anticipated attenuated progression to HF in δ-KO mice exposed to chronic VO. However, this was not clearly supported by the present study. Here we showed that CaMKIIδ deletion neither improved the survival pattern nor mitigated adverse myocardial remodeling, as evidenced by echocardiographic, gravimetric, histological, and molecular analyses, suggesting that induced CaMKII activity during early HF transition in long-term VO is secondary to the underlying pathologies.

Although PLB-Thr17 and RyR2-Ser2814 are endogenous substrates of CaMKII, phosphorylation at these sites were mildly decreased in δ-KO sham *vs*. WT sham. These results are consistent with previous reports^12,31^, and suggest that other kinases or CaMKII isoforms could compensate for phosphorylation at these sites. However, neither CaMKIIβ nor CaMKIIɣ showed any differential regulation upon CaMKIIδ deletion. Inconsistent with our data, Ling *et al*.^14^ have demonstrated massive decreased p-PLB-Thr17 and p-RyR2-Ser2814 levels in δ-KO mice. This discrepancy could be mice strain-dependent or due to different δ-KO strategies used. Here we used δ-KO mice generated via targeted deletion of exons 1–2 resulting in no translation of any residual CaMKII peptides^12^. In contrast, Ling *et al*.^14^ generated δ-KO mice by deletion of exons 9–11. Although they showed that CaMKIIδ was efficiently and completely deleted in δ-KO mice on the protein levels, the complete uncropped blots were not published and therefore we cannot rule out that the upstream exons, encoding the catalytic domains, would be translated giving a residual N-terminal peptide that might exert a dominant-negative effect on CaMKIIɣ and hence experiencing massive reduction of p-PLB-Thr17 and p-RyR2-Ser2814 levels that are comparable to that seen in CaMKIIδ/γ double KO mice^31^.

A striking observation of our study is the comparable CaMKII activity in δ-KO and WT hearts as inferred from CaMKII autophosphorylation. We can speculate that the induced CaMKII phosphorylation after shunt is due to increased CaMKIIγ activity, but not CaMKIIδ, suggesting that CaMKIIδ is entirely dispensable for VO-triggered adverse remodeling. One can also assume that CaMKIIδ deletion might be functionally compensated by CaMKIIγ. One of these assumptions might explain the lack of the beneficial effect of CaMKIIδ deletion on myocardial remodeling after shunt. Although we did not find an up-regulation of CaMKIIγ protein or mRNA in δ-KO mice, this does not exclude a possible role of CaMKIIγ activity in cardiac adaptation to VO. Indeed, Backs group has recently reported a redundant role of cardiac CaMKIIγ and CaMKIIδ isoforms in the context of PO and ischemia/reperfusion injuries^31,33^. Thus, further investigations are warranted to address whether CaMKIIγ-KO or CaMKIIδ/γ double KO mice would be protected against pathological remodeling triggered by VO.

Apoptosis is one of the pathophysiological features of maladaptive remodeling where it entails detrimental effects on cardiac contractility^34^. The CaMKIIδ has two isoforms generated by alternative splicing, viz, the anti-apoptotic nuclear δB and the pro-apoptotic cytosolic δC isoforms^30,35,36^. Here, we used δ-KO mouse model, lacking both δB and δC splice variants, and could show that cardiac apoptosis was similarly increased in shunt-operated δ-KO and WT hearts, suggesting that the anti-apoptotic effect of δC deletion could be buffered by pro-apoptotic effect of δB loss.

As opposed to the more extensively studied PO, there are no approved medical therapies to attenuate the adverse LV remodeling in the clinical VO of MR or AR because of limited VO experimental models available to efficiently mimic the chronic course of pure VO. This study was conducted using an established, reliable, and easily reproducible shunt model that induces pure VO, a condition that does not involve concomitant increases in PO, and therefore has helped to dissect the pathophysiology of VO. Although we consistently used 23-gauge needle to create the aorto-caval shunt, it is still difficult to control the shunt volume and we cannot rule out that shunt tends to distort, resulting in luminal stenosis and narrowing. However, LVEDD, SV and CO at 1-week post-surgery (early time point) were comparably increased in shunt-operated δ-KO and WT hearts, indicating a comparable shunt size and a similar degree of VO development in δ-KO and WT mice. Noteworthy, the severity of AR in human is classified according to the degree of SV induction and EF reduction, in which an increased SV by more than 65%, and a deteriorated cardiac function is correlated with severe degree AR that warrants surgical intervention. Interestingly, our shunt model experienced an increased SV by ≈70% and ≈63% at 1-week post-shunt in δ-KO and WT hearts, respectively, and a progressive EF deterioration. Therefore, we assume, that the degree of VO-triggered in our shunt model simulates the clinical situation seen in severe AR.

The VO is characterized by increased extracellular matrix turnover. Several reports showed a predominant inflammatory response immediately after shunt creation, which releases inflammatory mediators capable of activating matrix metalloproteinases that degrade collagen and therefore leads to eccentric hypertrophy and LV dilatation that even precedes cardiomyocytes elongation in VO model^37,38^. Consistently, both shunt-operated δ-KO and WT mice developed early LV dilatation but late functional deterioration. Beside systolic impairment, both shunt-operated groups experienced diastolic dysfunction as evidenced by deteriorated strain rate. Previously we and others have reported that VO induces an increased titin stiffness^2,39^ that could, on one side, limit eccentric hypertrophy and further myocardial dilatation. On the other side, increase titin-based sarcomere stiffness could limit active relaxation and passive distension and is therefore responsible for diastolic dysfunction seen in VO model.

The VO-triggered mortality is mainly due to cardiac dysfunction especially at long-term VO, when the cardiac function is impaired. But sudden cardiac death and fatal arrhythmias cannot be ruled out. Moreover, some mice would be presumably unable to compensate for the abrupt increase in preload and die of acute or subacute congestive heart failure at early phase VO. Noteworthy, shunt-operated mice exhibited higher mortality and severe remodeling than we have reported previously^3^, which is mainly due to strain and gender differences. While FVB/N mice show a mild myocardial remodeling following hemodynamic stress that takes longer time to develop HF, C57BL/6N line exhibits aggressive myocardial remodeling associated with higher mortality that progresses rapidly to congestive HF^40,41^. Moreover, males experience severe myocardial remodeling, associated with marked chamber dilatation and higher mortality. In contrast, females can better compensate for VO and show less dilatation and mortality^42^. In our previous study^3^ we used FVB/N female mouse line, whereas here we intentionally used both genders, to ensure reproducible data, in C57BL/6N substrain to investigate the effect of CaMKIIδ deletion on relatively severe form of VO-triggered myocardial remodeling.

In conclusion, we showed that, despite the reported relevance of CaMKII as a biomarker in HF patients, geometric, functional, and structural remodeling upon deletion of CaMKIIδ were not attenuated at early or late phases of VO. Our study not only excludes the critical role of CaMKIIδ in initiation or progression of adverse myocardial remodeling triggered by VO but also provides critical information for inhibition of CaMKIIδ as a potential therapeutic target for the maintenance of cardiac function in the setting of VO.

## Materials and Methods

### Mice

All investigations were approved by the responsible Institutional Review Board (Lower Saxony State Office for Consumer Protection and Food Safety (LAVES), conforms to the Guide for the Care and Use of Laboratory Animals (NIH publication No. 85-23, revised 1996), and was performed in accordance with the ethical standards laid down in the Declaration of Helsinki 1964. As previously described, the CaMKIIδ-KO mice^12^ were generated by breeding floxed CaMKIIδ with CAG-Cre transgenic mice^43^ to give heterozygous CaMKIIδ^+/−^ mice that were intercrossed to each other to obtain global homozygous CaMKIIδ^−/−^ mice (δ-KO). In this study, we analyzed male and female δ-KO and WT littermates on C57BL/6N genetic background.

### Aortocaval shunt

Shunt surgery was done as previously described^44^. Briefly, 8-week-old δ-KO and WT littermates were anesthetized using isoflurane insufflation and a longitudinal abdominal incision was made to expose the infrarenal abdominal aorta and inferior vena cava. A 23-gauge needle was inserted into the infrarenal aorta at a 45degree angle and passed through to the vena cava, creating the shunt. After needle withdrawal, the aortic puncture was sealed using cyanoacrylate (Pattex, Düsseldorf, Germany). In successful shunt creation, pulsatile flow of oxygenated blood from the infrarenal aorta into the inferior vena cava could be visualized. The abdomen was then closed, and the mice were kept on a heating plate until full recovery from anesthesia. Sham animals underwent the same procedure except for the puncture of the vessels.

### Transthoracic echocardiography

The mice were anesthetized using 1.5% isoflurane, and echocardiography was performed using a VS-VEVO 660/230 (Visual Sonics, Toronto, Canada). 2D guided M-mode images were recorded in the long-axis view at the left mid-ventricular level. Strain analysis was performed as described recently^45^. The Echo measurements and analyses were carried out by 2 independent scientists who were completely blind towards group assignment.

### Histological analysis

The hearts were fixed in 4% buffered formaldehyde overnight, paraffin-embedded, sectioned (5 µm), and stained with hematoxylin-eosin (H&E) for cell morphology. For minimal cardiomyocyte fiber diameter, sections were stained with fluorescein-conjugated wheat germ agglutinin (WGA-Alexa Fluor 594, Invitrogen, Carlsbad, CA, USA) and were then quantified using ImageJ software (Bethesda, MD, USA). At least 500 randomly selected transversely cut myocytes from 4 animals/group were measured. Picrosirius red staining was performed and quantified on sections using ImageJ software. TUNEL assays were performed with the *In-Situ* Cell Death Detection Kit (Roche Molecular Biochemicals, Mannheim, Germany) according to manufacturer’s instructions. TUNEL-positive cells were counted in 5–8 random 20X fields per heart from 4–5 mice/group.

### Western blot analysis

Frozen LV was homogenized in RIPA buffer (Millipore, Schwalbach am Taunus, Germany) containing protease and phosphatase inhibitor cocktail tablets (Roche, Mannheim, Germany). Extracted proteins (20 µg) were subjected to sodium dodecyl sulfate polyacrylamide gel electrophoresis (SDS-PAGE) and blotted onto a nitrocellulose or Polyvinylidene Difluoride (PVDF) membranes (Bio-Rad, München, Germany). Membranes were blocked for 1 h with 5% milk in TBS-Tween at room temperature and then incubated with the following primary antibodies: Rabbit polyclonal anti-phospho-Ser2814-RyR2 (Badrilla, Leeds, UK), anti-phospho-Thr-17 Phospholamban (Badrilla), anti-RyR2 (Sigma-Aldrich, Munich, Germany), anti-CaMKIIγ and anti-CaMKIIδ (Thermo Fisher, Bonn, Germany), and mouse monoclonal anti-phospho-CaMKII (Affinity BioReagents, Golden, CO, USA), anti-Phospholamban (Millipore, Hamburg, Germany), anti-Serca2a (Affinity BioReagents), and anti-GAPDH (Santa Cruz Biotechnology, Santa Cruz, CA, USA). Blots were subsequently incubated with horseradish peroxidase (HRP)-conjugated secondary antibodies, and finally were detected using an enhanced chemiluminescent detection system (Amersham Bioscience, Braunschweig, Germany) according to the manufacturer’s instructions. For quantification, band intensity was normalized to total protein load obtained from Ponceau-stained blot using Image Lab software (Bio-Rad).

### Quantitative real time polymerase chain reaction (qRT-PCR)

Using the RNeasy kit (Qiagen, Hilden, Germany), RNA was extracted from the LV, and cDNA was synthesized by the iScript cDNA synthesis kit (Bio-Rad). QRT-PCR was performed on a Bio-Rad iQ-Cycler using SYBR Green Supermix (Bio-Rad). Used primer sequences were:

**Table:** 

*Gapdh*	sense:	GAGACGGCCGCATCTTCTT
	antisense:	CAATCTCCACTTTGCCACTGC
*Nppa*	sense:	GGGGGTAGGATTGACAGGAT
	antisense:	CAGAATCGACTGCCTTTTCC
*Nppb*	sense:	GCACAAGATAGACCGGATCG
	antisense:	CTTCAAAGGTGGTCCCAGAG
*Serca2a*	sense:	GGGCAAAGTGTATCGACAGG
	antisense:	TCAGCAGGAACTTTGTCACC
*CaMKIIα*	sense:	GCTGCCAAGATTATCAACACC
	antisense:	CACGCTCCAGCTTCTGGT
*CaMKIIβ*	sense:	GCCATCCTCACCACTATGCT
	antisense:	CTCCATCTGCTTTCTTGTTGAGT
*CaMKIIɣ*	sense:	AGTTCACAGGGACCTGAAGC
	antisense:	CGCCTTGAACTTCTATGGCTA
*CaMKIIδ*	antisense:	GTGCCATCCTCACAACCAT
	antisense:	CATCTGACTTCTTGTTCAATAGGC

### Statistical analysis

Statistical analyses were performed with GraphPad Prism 7.0 (GraphPad Software, Inc, California, USA) with two-tailed unpaired Student’s t-test or one-way ANOVA with Bonferroni post-test correction where appropriate. Kaplan–Meier survival analysis was performed, and a Log-rank test was used to determine significance. Data are expressed as mean ± SEM (standard error of the mean), and a *p* < 0.05 was considered statistically significant.

## Supplementary information

Supplementary Materials

## Data Availability

All data generated or analyzed during this study are included in the published article and the Supplementary Materials.
