# Supplementary material for: Genetic deletion of calcium/calmodulin-dependent protein kinase type II delta does not mitigate adverse myocardial remodeling in volume-overloaded hearts
Source: Sci Rep. 2019 Jul 8;9:9889. doi: 10.1038/s41598-019-46332-3 (PMC6614357; doi:10.1038/s41598-019-46332-3)
Supplement: Supplementary file 1 — Supplementary Materials [file 41598_2019_46332_MOESM1_ESM.pdf]

Supplementary Materials for

Genetic deletion of calcium/calmodulin-dependent protein kinase type II delta does not mitigate adverse myocardial remodeling in volume-overloaded hearts

Belal A. Mohamed, Manar Elkenani, Joanna Jakubiczka-Smorag, Eric Buchholz, Sabrina Koszewa, Dawid Lbik, Moritz Schnelle, Gerd Hasenfuss, Karl Toischer

The file includes:

Figure S1: Full-length blots of the cropped blots displayed in Figure 6.

Table S1: Basal echocardiographic parameters in δ-KO and WT littermates at 2 months of age.

Figure S1: Full-length blots of the cropped blots displayed in Figure 6.

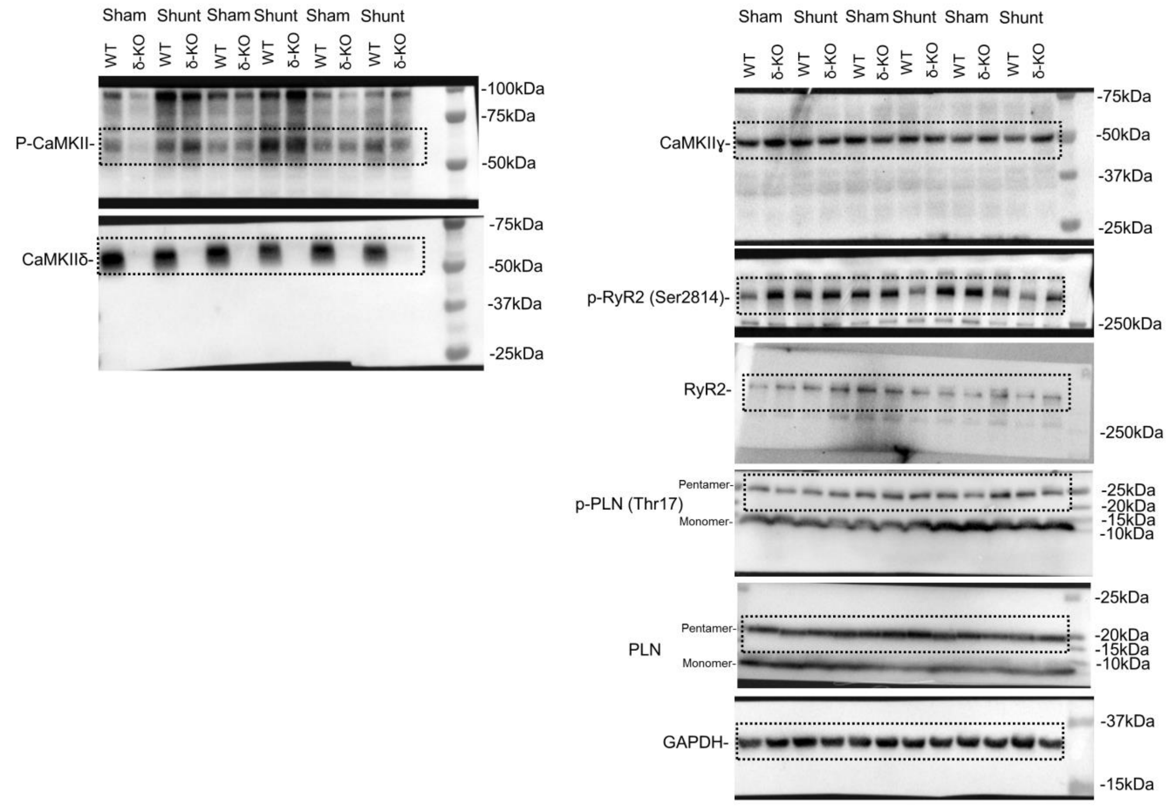

Table S1 Basal echocardiographic parameters in δ-KO and WT littermates at 2 months of age.

	WT (*n*= 6)	δ-KO (*n*= 5)
HR (bpm)	403.7 ± 9.71	420.4 ± 17.09
LVESD (mm)	3.37 ±0.14	3.42 ± 0.20
LVEDD (mm)	4.27 ± 0.15	4.19 ± 0.15
EF (%)	49.64 ± 2.58	48.36 ± 2.42
FS (%)	21.49 ± 2.49	22.11 ± 1.29
Septum (mm)	0.66 ± 0.04	0.61 ± 0.03
LVM/EDV (mg/µl)	1.36 ± 0.09	1.30 ± 0.08

Data are presented as mean ± SEM. Two-tailed unpaired Student's *t*-test. bpm beats per minute; EF, ejection fraction; HR, heart rate; LVEDD, left ventricular end-diastolic diameter; LVESD, left ventricular end-systolic diameter; LVM, left ventricle mass; SV, stroke volume; WT, wild-type.
